# Improvement and efficient display of *Bacillus thuringiensis* toxins on M13 phages and ribosomes

**DOI:** 10.1186/s13568-015-0160-1

**Published:** 2015-11-25

**Authors:** Sabino Pacheco, Emiliano Cantón, Fernando Zuñiga-Navarrete, Frédéric Pecorari, Alejandra Bravo, Mario Soberón

**Affiliations:** Instituto de Biotecnología, Universidad Nacional Autónoma de México, Apdo. postal 510-3, Cuernavaca, 62250 Morelos Mexico; Institut de Recherche en Santé de l’Université de Nantes, INSERM U892, CNRS, 6299-CRCNA, 8 quai Moncousu, BP 70721, 44007 Nantes Cedex 1, France

**Keywords:** *Bacillus thuringiensis*, Phage display, Ribosome display, Cry toxins

## Abstract

*Bacillus thuringiensis* (Bt) produces insecticidal proteins that have been used worldwide in the control of insect-pests in crops and vectors of human diseases. However, different insect species are poorly controlled by the available Bt toxins or have evolved resistance to these toxins. Evolution of Bt toxicity could provide novel toxins to control insect pests. To this aim, efficient display systems to select toxins with increased binding to insect membranes or midgut proteins involved in toxicity are likely to be helpful. Here we describe two display systems, phage display and ribosome display, that allow the efficient display of two non-structurally related Bt toxins, Cry1Ac and Cyt1Aa. Improved display of Cry1Ac and Cyt1Aa on M13 phages was achieved by changing the commonly used peptide leader sequence of the coat pIII-fusion protein, that relies on the Sec translocation pathway, for a peptide leader sequence that relies on the signal recognition particle pathway (SRP) and by using a modified M13 helper phage (Phaberge) that has an amber mutation in its pIII genomic sequence and preferentially assembles using the pIII-fusion protein. Also, both Cry1Ac and Cyt1Aa were efficiently displayed on ribosomes, which could allow the construction of large libraries of variants. Furthermore, Cry1Ac or Cyt1Aa displayed on M13 phages or ribosomes were specifically selected from a mixture of both toxins depending on which antigen was immobilized for binding selection. These improved systems may allow the selection of Cry toxin variants with improved insecticidal activities that could counter insect resistances.

**Electronic supplementary material:**

The online version of this article (doi:10.1186/s13568-015-0160-1) contains supplementary material, which is available to authorized users.

## Introduction

*Bacillus thuringiensis* (Bt) produces insecticidal Cry proteins that kill important crop pests and also mosquitoes that are vectors of human diseases (Bravo et al. 2011; Pardo-López et al. 2013). Bt strains produce different non-related Cry toxins including the 3-domain family of Cry toxins (3D-Cry) such as Cry1Ac or the Cyt family of toxins such as Cyt1Aa. Members of the 3D-Cry family show insect specificity to dipteran (ex. Cry4Ba), coleopteran (ex. Cry3Aa) and lepidopteran (ex. Cry1Ac) insects. It is widely accepted that 3D-Cry toxins exert their toxic effect by the sequential binding to insect gut proteins named cadherins and glycosyl-phosphatidyl-inositol (GPI) anchored proteins such as aminopeptidase-N (APN) or alkaline phosphatase (ALP) resulting in toxin oligomerization and pore formation that finally cause cell lysis and disruption of the insect gut (Pardo-López et al. 2013).

Most Bt strains that show toxicity against dipteran insects also produce a different family of insecticidal proteins called Cyt toxins (Soberón et al. 2013). Both Cry and Cyt toxins are synthesized as protoxins that are activated by insect gut proteases releasing active toxin fragments (Pardo-López et al. 2013). Cyt toxins are also pore-forming toxins, that in contrast to Cry toxins, does not interact with protein receptors binding directly to membrane lipids present in insect gut cells, resulting in membrane insertion (Soberón et al. 2013). More interestingly, Cyt1Aa synergizes the insecticidal activity of the 3D-Cry Cry11Aa and Cry4Ba toxins by their binding interaction, functioning as a surrogate receptor molecule (Pérez et al. 2005; Cantón et al. 2011). Recently it was shown that Cyt2Aa specificity could be modified to kill aphids by the insertion of a peptide sequence, in exposed loop regions, that mediates binding to an aphid APN (Chougule et al. 2012).

The molecular evolution of Bt toxins could provide improved toxins against insect species that are poorly controlled by the available Cry toxins and also for the selection of Bt toxins that could recover toxicity to insects that develop resistance to the action of these proteins (Bravo et al. 2013). Different display systems that allow construction of libraries of mutants and selection of binders with improved affinity, along with the gene coding for these variants, have been developed such as phage display or ribosome display systems (Hanes and Plückthun 1997; Bratkovic 2010).

Phage display system allows selection of protein variants with improved binding characteristics (Azzazy and Highsmith 2002; Mullen et al. 2006). For this purpose, the foreign protein DNA sequence is fused to a coat protein gene enabling the fusion protein to be displayed on the surface of the phage that can be then screened by enabling the phage to interact with ligands, a process known as biopanning allowing the molecular evolution of proteins (Dröge et al. 2003; Mullen et al. 2006). To display the fusion protein, it has to be translocated to the host periplasm by a peptide leader sequence while a helper phage provides all the necessary components for phage assembly. In contrast, the ribosome display system involves an in vitro translation of the protein that prevents the synthesized protein and the mRNA from leaving the ribosome and binders with improved affinities are selected by panning (Hanes and Plückthun 1997). Ribosome-display allows construction of libraries with sizes up to 10^14^ in contrast with phage display where libraries of 10^9^ size are typically obtained due to the limitation in *E. coli* transformation efficiency (Dreier and Pluckthun 2011).

Cry1A toxins have been displayed in three different phages (M13, T7 and λ), these systems have shown different problems for displaying Cry toxins (Marzari et al. 1997; Kasman et al. 1998; Vílchez et al. 2004; Pacheco et al. 2006). In the case M13, the Cry1Aa and Cry1Ac toxins were not properly displayed resulting in deletions of the Cry1Aa-fused protein, while the displayed Cry1Ac protein did not bind to functional receptors in vitro suggesting structural constraints of the toxin (Marzari et al. 1997; Kasman et al. 1998). In contrast the Cry1Ac toxin was efficiently displayed in both T7 and λ phages where the assembling of these phage particles occurs in the cytoplasm (Vílchez et al. 2004; Pacheco et al. 2006). Nevertheless, T7 and λ systems rely on in vitro packaging systems that are quite labile and have limitations for the construction of libraries with large number of variants with a limit up to 10^7^ to 10^8^ variants.

Improvement of M13 display can be achieved by using engineered helper phages such as the “Phaberge” helper phage that contains an amber mutation on its genome favoring the assembling of phage particles containing the recombinant pIII-fusion protein when non-suppressive *E. coli* host cells were infected (Soltes et al. 2003). Also, improvement of display of several different proteins on M13 was achieved by changing the commonly used peptide leader sequence of the fusion protein, that relies on the Sec translocation pathway, for a peptide leader sequence that relies on the signal recognition particle pathway (SRP) (Steiner et al. 2006). The Sec and SPR pathways translocate proteins to the periplasm post-translationally and co-translationally, respectively (Steiner et al. 2006). Contrary to phage display, ribosome display is a free-cell system that does not require in vivo processes to display the proteins; therefore the environment for production, folding and stability of the interest-protein can be manipulated and optimized in vitro. These features have been exploited to select and evolve peptides, proteins and antibody fragments (Wörn and Plückthun 2001; Jermetus et al. 2001; Osada et al. 2009; Mouratou et al. 2007; Correa et al. 2014).

In this work, we have adapted and/or improved these two complementary systems with the aim to maximize chances to have an efficient display whatever the family of Cry toxins to evolve. We show the successful display of Cry1Ac and Cyt1Aa toxins on M13. Also, Cry1Ac and Cyt1Aa were successfully displayed using ribosome-display. The two Bt toxins retain their specific binding characteristics for their specific ligands. Furthermore, we show that specific selection of the displayed toxins by biopanning. These improved systems may allow the selection of Cry toxin variants with improved insecticidal activity that could counter insect resistances.

## Materials and methods

### Molecular biology

Enzymes, buffers and mass weight marker were from Fermentas, Thermo Scientific. All PCRs were performed with Phusion DNA-polymerase with HF buffer. Primers sequences are provided at Table 1.

**Table 1:** List of primers

Primer	Sequence (5′→3′)	Restriction site
SP1	AGAGCATGCGTAGGAGAAAATAAAATGAAAAAGATTTGGCTGGCGCTGGCTGG	–
SP2	TCTTTGTAGTCCGCCGATGCGCTAAACGCTAAAACTAAACCAGCCAGCGCCAGCC	–
SP3	CCCCAAGCTTGGGAGAGCATGCGTAGGAG	*Hin*d III
SP4	(GA)_10_ GGCCGGCTGGGCCCCTTTGTAGTCCGCCG	*Sfi* I
SupASC	GGAACCGCGTGCCGCACAGACTGTTGAAAGTTG	–
FFTcM13	(GA)_10_ GGCCCAGCCGGCCGGAGAAAGAATAGAAACTGG	*Sfi* I
RFTcM13	ATAGTTTAGCGGCCGCTGGAATAAATTCAAATCTGTC	*Not* I
FDIIc	TCACGGATCCAGAGAAATTTATACAAACCCAGTATTAGAAAAT	*Bam* HI
RDIIc	TATAAAGCTTACTACGATGTATCCAAGAGAACATAGGAGCTCTTATTATACT	*Hin*d III
3Aup	(GA)_10_ GGCCCAGCCGGCCAAAGATGTCATTCAAAAAGGC	*Sfi* I
3Alow	(GA)_10_ GCGGCCGCATTCACTGGAATAAATTCAATTTTGTC	*Not* I
Pro-Sf1	(GA)_10_ GGCCCAGCCGGCCATGGAAAATTTAAATCATTGTCC	*Sfi* I
Pro-Not	ATAGTTTAGCGGCCGCGAGGGTTCCATTAATAGCGC	*Not* I
T7B	ATACGAAATTAATACGACTCACTATAGGGAGACCACAACGG	–
SDMRGS	AGACCACAACGGTTTCCCTCTAGAAATAATTTTGTTTAACTTTAAGAAGGAGATATATCCATGAGAGGATCG	–
TolAkurtz	CCGCACACCAGTAAGGTGTGCGGTTTCAGTTGCCGCTTTCTTTCT	–
FFTc	TCACGGATCCGGAGAAAGAATAGAAACTGG	*Bam* HI
RFTc	TATAAAGCTTTGGAATAAATTCAAATCTGTC	*Hin*d III
CytFRD	CACGGATCCCCAAATGAAATCAATAATCTTC	*Bam* HI
CytRRD	ATAAAGCTTTGGTTGTGCAAATTTCAAAGATTG	*Hin*d III

Sequences of the restriction sites are underlined

### Preparation of Bt toxins and cadherin fragment CR7-12

Crystals of Cry1Ac, Cry11Aa, Cry3Aa and Cyt1Aa were produced from *Bacillus thuringiensis*, purified, solubilized and activated as previously reported (Gómez et al. 2001; Pérez et al. 2005). Cadherin fragment CR7-12 was expressed in *E. coli* ER2566 and purified with nickel affinity column as reported (Pacheco et al. 2009).

### Phagemids construct and cloning

The signal sequence PelB of the pCANTAB 5E phagemid was substituted by ssDsbA to construct pCAD phagemid. The ssDsbA was synthetized by overlapping-PCR of four primers as previously reported (Steiner et al. 2006). Briefly, 10 pmol of SP1 and SP2 primers were mixed and assembled at 72 °C for 10 min with Phusion DNA-polymerase, then 1 μl of the assembly reaction was used as template and amplified with SPIII and SP4 primers. The PCR product was separated by agarose-gel electrophoresis and ligated into pCANTAB 5E after restriction with *Hin*dIII and *Sfi*I enzymes. Phagemid pCADS was constructed by mutagenizing the TAG amber stop codon of pCAD phagemid by CAG codon with the primer SupASC using the QuikChange II Site-Directed Mutagenesis Kit (Agilent Technologies) following the manual instructions. The *cry1Ac* toxic fragment coding-sequence (G26-A614) (GenBank No. AM949588) was amplified by PCR from *Bacillus thuringiensis* strain HD73 (Bacillus Genetic Stock Center-BGSC- No. 4D4) using the primers FFTc-M13/RFTc-M13. The *cyt1Aa* protoxin fragment coding-sequence (GenBank No. X03182) was amplified by PCR from pWF45 vector (Wu and Federici, 1993) using the primers Pro-SfiI/Pro-NotI. The *cry3Aa* toxic fragment coding-sequence (K63-N644) (GenBank No. AJ237900) was amplified by PCR from *Bacillus thuringiensissubsp. tenebrionis* (BGSC No. 4AA1) using the primers 3Aup/3Alow. All the PCR-amplified toxin fragments were ligated after digestion with *Sfi*I and *Not*I restriction enzymes on previously digested pCANTAB 5E, pCAD or pCADS phagemids.

### Phage production and purification

The pCANTAB 5E and pCADS phagemids with the *cry1Ac* toxin gene inserted were transformed into *E. coli* XL1-Blue MRF’, while pCADS with *cry1Ac* and *cyt1Aa* toxin genes were transformed into *E. coli* HB2151. A single colony was grown overnight at 37 °C in 3 ml of 2xYT medium containing 100 μg/ml ampicillin. The overnight culture was used to inoculate 35 ml of 2xYT containing 100 μg/ml ampicillin and were incubated at 37 °C until reaching an OD_600_ of 0.6. The *E. coli* XL1-Blue MRF’ or HB2151 cultures were infected with 10^11^ particles of helper phages VCSM13 or Phaberge (which was kindly donated by Dr. Erik J. Wiersma), respectively. After 30 min of incubation at room temperature, kanamycin (30 μg/ml) was added and the cultures were incubated at 30 °C overnight. The supernatants were separated by centrifugation and 6 ml of PEG-NaCl solution (20 % PEG 8000/2.5 M NaCl) was added and incubated at 4 °C for 1 h. The phages were precipitated by centrifugation at 8500*g* for 10 min at 4 °C and the pellet was suspended in 1 ml of PBS pH 7.4. Finally the phage titer was estimated counting colonies resistant to ampicillin after *E. coli* XL1-Blue MRF’ infection.

### Phage blot

10^11^ phage particles were mixed in Laemmli buffer and boiled for 5 min. The mixture was separated by SDS-PAGE and then electrotranferred to PVDF Immobilon-P membrane (Millipore). After blocking with PBS-M (PBS, 5 % skim milk) for 1 h, the membrane was incubated with polyclonal rabbit antibodies anti-Cry1Ac or anti-Cyt1Aa (1:20,000 in PBS, 0.1 % Tween-20). The membrane was incubated for 1 h with the secondary antibody anti-rabbit IgG-HRP (Santa Cruz Biotechnology) diluted to 1:30,000 in PBS-T. Finally, blots were visualized with Western Blotting Luminol Reagent (Santa Cruz Biotechnology) and densitometry was performed with ImageJ software (http://imagej.nih.gov/ij/).

### Phage binding

ELISA 96-well microplates were coated overnight at 4 °C with 100 µl of either cadherin protein fragment CR7-12 (200 nM), anti-Cry1Ac antibody (1:1000) or anti-Cyt1Aa antibody (1:1000) diluted in PBS. The plates were blocked with 200 µl/well of PBS-M for 1 h at 37 °C. Phages particles (10^10^) per well diluted in PBS-T were added and incubated for 1 h, then the unbound phages were removed by washing with PBS-T. The anti-pVIII-HRP secondary antibody (1:5000) was added and incubated for 1 h. The HRP enzymatic activity was revealed with 100 µl of substrate solution (1 mg/ml ortho-phenylenediamine, 0.05 % H_2_O_2_, 100 mM NaH_2_PO_4_ pH 5.0). Reaction was stopped adding 50 µl of 1 M H_2_SO_4_ and measured at 490 nm using the Emax Precision Microplate Reader, Molecular Devices.

For competition binding assay, M13-Cry1Ac phages were incubated in presence of increasing concentrations of soluble Cry1Ac toxin and the phage binding was performed as described above.

### Insect bioassay

*Manduca sexta* neonate larvae were reared on an artificial diet in 24-well plates. Cry1Ac activated toxins (10 ng/cm^2^), M13-Cry1Ac or M13 wild-type phages (10^11^ pfu/cm^2^) were applied on the surface of diet and one neonate larvae was placed per well. Mortality was recorded after 7 days at 28 °C, 65 ± 5 % relative humidity and a photoperiod of 16/8 h light/dark. Mortality was scored after 7 days.

### Phage selection

ELISA 96-wells plates were coated with the bait antigen (CR7-12, anti-Cry1Ac or anti-Cyt1Aa) as described for phage binding. A mixture 1:1 of M13-Cry1Ac or M13-Cyt1Aa (10^10^ pfu/100 μl) in PBS-T was added per well and incubated for 1 h at room temperature with gentle shaking. The wells were washed ten times with PBS-T and 100 μl of E. coli XL1-Blue MRF’ grown in 2xYT medium until OD_600_ 0.6 were added. The plates were incubated for 30 min at room temperature to allow the infection and the cells were collected and diluted in 1.4 ml of a 2xYT medium. An aliquot of 50 μl of cells diluted were plated on agar LB plates containing ampicillin 100 μg/ml and were incubated at 37 °C for 16 h. To determine which phages were selected depending on bait was used as antigen, colony-PCR was performed to identify the phagemid by amplifying either the *cry1Ac* or *cyt1Aa* genes with the primers FFTc-M13/RFTc-M13 or Pro-SfiI/Pro-NotI, respectively. The PCR-products were evaluated by agarose gel electrophoresis.

### In vitro transcription and translation

To prepare the mRNA of Cry1Ac (G26-P607), domain II of Cry1Ac (Q262-S459) or Cyt1Aa (P34-P229), the gene fragments were PCR amplified with primers FFTc/RFTc, FDIIc/RDIIc or CytF-RD/CytR-RD, respectively. After restriction with *Bam*HI and *Hin*dIII enzymes, the PCR fragments were inserted into pFPRDV plasmid. To obtain the DNA template for in vitro transcription, the outer primers T7B and TolAkurtz were used to introduce, by PCR amplification, T7 promoter, ribosome binding site and TolA spacer sequences. The PCR products were used as DNA template for in vitro transcription using TranscriptAid T7 High Yield Transcription Kit (Fermentas, Thermo Scientific).

For in vitro translation, S30 extract were prepared as described previously (Dreier and Pluckthun 2011). Briefly, *E. coli* MRE600 were grown in rich medium (40 mM KH_2_PO_4_, 165 mM K_2_HPO_4_, 10 g/L yeast extract, 15 μg/ml thiamine, 2 % glucose, 1 mM MgOAc) until DO_600_ of 1.0. Cells were washed three times with buffer S30 (14 mM MgOAc, 60 mM KOAc, 10 mM Tris-OAc pH 7.5) and lysed with French press. Afterward, the lysate was clarified by centrifugation at 30,000 g and then it was dialyzed overnight with buffer S30. The ternary complex was prepared in a translation reaction of 27.5 μl containing 2.5 μg of mRNA, 12.5 μl of S30 extract and 11 μl of PremixZ (Dreier and Pluckthun 2011). The reaction was incubated at 37 °C for 15 min and then was placed on ice. To maintain the ternary complex, 75 μl of WB (50 mM Tris–Acetate pH 7.5, 150 mM NaCl, 50 mM Magnesium Acetate) were added; to dissociate the ternary complex, 75 μl of TBS were added.

### ELISA test of the in vitro translated toxins

ELISA microplates coated with the bait antigen were blocked with TBS-BSA 0.5 %. An in vitro translation reaction prepared with *cry1Ac* or *cyt1Aa* mRNA and stopped with TBS, was applied and incubated for 1 h with gentle shaking at room temperature. Binding of Cry1Ac against cadherin fragment CR7-12 was detected with the polyclonal antibody anti-Cry1Ac followed of secondary antibody anti-rabbit IgG-HRP. In the case of immobilized antibodies anti-Cry1Ac or anti-Cyt1Aa the binding was detected with an antibody anti-RGS-His-HRP. HRP enzymatic activity was revealed as was described above.

### Recovery of RNA after binding selection of ternary complexes

ELISA microplates coated with the bait antigen (CR7-12, Cry11Aa or anti-Cyt1Aa) were blocked with TBS-BSA 0.5 % and washed with WB. An in vitro translation reaction prepared with an equimolar mixture of Cry1Ac and Cyt1Aa mRNA and stopped with WB, was applied and the plate was incubated 1 h with gentle shaking at 4 °C followed by ten washes steps with WB-T. The mRNA was eluted with 100 µl of elution buffer (50 mM Tris-HOAc pH 7.5, 150 mM NaCl, 10 mM EDTA, 5 µg *Saccharomyces cerevisiae* RNA) during 10 min at 4 °C with gentle shaking and purified with RNeasy kit (Qiagen). Reverse transcription was performed with RevertAid RT Kit (Fermentas, Thermo Scientific) according to manual instructions. The cDNA obtained from reverse transcription with the reverse primers RFTc or CytR-RD was PCR-amplified with the primers SD-MRGS/RFTc or SD-MRGS/CytR-RD, respectively. Finally, the PCR products were analyzed by agarose gel electrophoresis.

## Results

### Constructs for the display of Bt toxins on phages

**Fig. 1:**
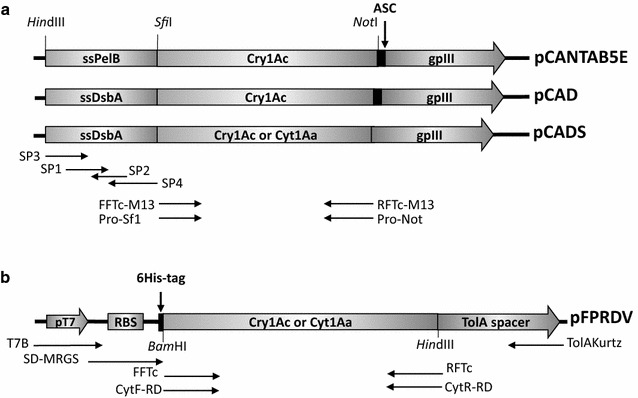
**a** Schematic representation of the phagemid constructs for phage display. *ASC* amber stop codon. **b** Schematic representation of the constructs for ribosome display. RBS, ribosome binding-site. *Horizontal arrows* indicate the annealing region of the primers at the constructs

With the aim to improve the phage display of Bt toxins we compare the display efficiency of Bt toxins on M13 phage by using the secretory Sec or SRP pathways. We designed a phagemid for the fusion of Bt toxins to pIII viral protein that depends on a SRP signal sequence. The ssDsbA, which tags the proteins to its SRP-dependent translocation, was generated by assembly-PCR as previously reported (Steiner et al. 2006). The pCANTAB 5E phagemid was used as backbone vector (Amersham Biosciences) and the ssPelB was substituted by ssDsbA, hereafter called pCAD (Fig. 1a). In addition, to increase the display levels of toxin by using a helper phage with a conditional deficiency of pIII production (phaberge), the amber stop codon of pCAD between the toxin-pIII fusion was mutagenized (pCADS vector).

### SRP pathway increases the display level of functional Bt toxins

**Fig. 2:**
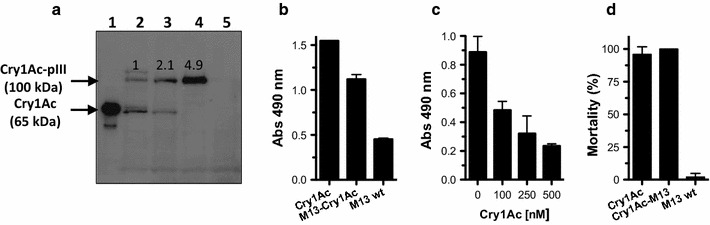
**a** Western blot analysis of 10^11^ M13 phages displaying Cry1Ac toxin prepared from *E. coli* cells harbouring the phagemids pCANTAB 5E-Cry1Ac (*lane 2*), pCAD-Cry1Ac (*lane 3*) or pCADS-Cry1Ac (*lane 4*). *Lane 1* is trypsin-activated Cry1Ac toxin and lane 5 is the M13 helper phage. Values above the *bands* indicate the number fold of Cry1Ac displaying level as judged by the optical density of the 100 kDa band. **b** ELISA binding assays of M13-Cry1Ac phage particles, prepared from the phagemid pCAD-Cry1Ac, to cadherin fragment CR7-12. **c** Analysis of binding competition by ELISA of M13-Cry1Ac in presence of increasing concentrations of Cry1Ac toxin. **d** Toxicity of Cry1Ac-M13 (10^11^) to neonates *M. sexta* larvae after 7 days. *Error bars* at each plot represent the standard deviation

The display efficiency by Sec and SPR translocation of the toxic fragment of Cry1Ac toxin (G26-A614) were compared. M13 phages were prepared from *E. coli* cells harboring pCANTAB 5E-Cry1Ac or pCAD-Cry1Ac vectors. The same amount of phage particles (10^11^) were subject to SDS-PAGE and the fusion protein Cry1Ac-pIII was revealed by western blot with anti-Cry1Ac polyclonal antibody. Figure 2a shows that the M13 phages prepared with pCAD produced two fold more of the fusion Cry1Ac-pIII protein than M13 phages prepared from pCANTAB 5E as judged by scanning the optical density of the 100 kDa band.

The binding of Cry1Ac-M13 phages to cadherin fragment from domain repeats CR7 to CR12 of *M. sexta* (M810-A1485), which is a functional receptor for Cry1Ac (Pacheco et al. 2009), was analyzed. Figure 2b shows an ELISA binding assay showing that M13-Cry1Ac prepared from *E. coli* cells harboring the phagemid pCAD-Cry1Ac bound to CR7-12. An ELISA competitive assay showed that the binding of virions M13-Cry1Ac to CR7-12 was specific since it was competed by Cry1Ac toxin (Fig. 2c). The toxicity of the virions Cry1Ac-M13 was analyzed in bioassays by diet surface exposure showing that Cry1Ac-M13 was toxic to *M. sexta* larvae indicating that the display toxin retains its insecticidal activity (Fig. 2d). Together these results show that the display Cry1Ac toxin using the ssDsbA retains its receptor binding and toxicity features suggesting no structural constrains in the toxin displayed.

It is well established that pIII-deficient helper phages increase the amount of exogenous protein displayed on M13 (Soltes et al. 2003). Phaberge is a helper phage with an amber stop codon in pIII gene, therefore non-SupE *E. coli* strains reduce the amount of non-fused pIII production and most is provided for phage assembly by the phagemid containing the pIII fused protein. To test if Phaberge increases the level of Cry1Ac-pIII incorporated to the capsid, we used the non-SupE *E. coli* HB2151 strain housing the pCADS-Cry1Ac vector and M13 phage were produced. Western-blot analysis of the viral particles showed more than two fold increased production of Cry1Ac-pIII protein compared with the same construct using M13 phage (Fig. 2a, lanes 3 and 4).

**Fig. 3:**
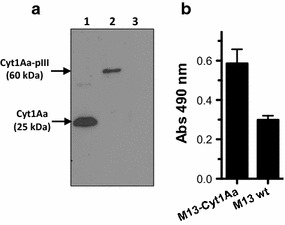
**a** Western blot analysis of M13 phage particles displaying Cyt1Aa toxin prepared from *E. coli* cells harbouring the phagemid pCADS-Cyt1Aa. *Lane 1* Cyt1Aa protoxin; *lane 2* M13-Cyt1Aa phage particles; lane 3, M13 helper phage. **b** ELISA binding assay of M13-Cyt1Aa to immobilized polyclonal anti-Cyt1Aa antibody.* Error bars* represent the standard deviation

In order to test if Cyt1Aa toxin can be displayed on M13 phage, we cloned Cyt1Aa protoxin fragment in pCADS vector. Phages prepared with this construct were analyzed as was described for M13-Cry1Ac. Figure 3a shows a phage blot assay showing that Cyt1Aa protoxin is displayed on filamentous phages. Figure 3b shows that the phages M13-Cyt1Aa are able to bind to Cyt1Aa antibody.

### Ribosome display of Bt toxins

**Fig. 4:**
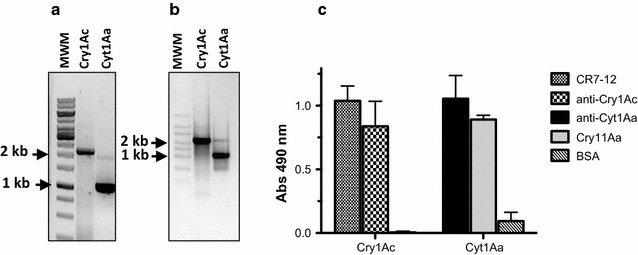
**a** DNA template prepared by PCR amplification for ribosome display of Cry1Ac and Cyt1Aa toxins. *MWM* Mass weight marker GeneRuler 1 kb DNA Ladder. **b** mRNA of Cry1Ac and Cyt1Aa obtained from in vitro transcription. MWM, Mass weight marker RiboRuler 6000 RNA Ladder **c** ELISA binding assay of the nascent Cry1Ac or Cyt1Aa toxin from ribosomal complex

In contrast to phage display, ribosome display is a fully in vitro method for molecular display. To explore the ribosome display as alternative display system, Bt toxins genes were cloned in the pFPRDV vector (Correa et al. 2014). This vector enables the incorporation of T7 promoter and ribosome binding-site sequences for transcription and translation in vitro, respectively; also the fusion of TolA spacer at C-terminal of the toxins without stop-codon is included in pFPRDV (Fig. 1b). The toxic fragments of Cry1Ac or Cyt1Aa toxins were PCR amplified from the constructs of pFPRDV using the primers T7B and TolAkurtz as described in materials and methods (Fig. 4a). The PCR product contains all necessary elements for ribosome display: the T7 promoter for in vitro transcription with T7 polymerase, the canonical Shine-Delgarno ribosome binding-site sequence for an efficient translation with S30 extract prepared from *E. coli* and the toxin-coding sequence fused to TolA spacer, which remains anchored into the ribosomal tunnel at the ternary complex. The fragments obtained by PCR were in vitro transcribed and analyzed by agarose gel electrophoresis. Figure 4b shows the mRNA bands with the expected molecular weight of approximately 1850 bp for *cry1Ac* or 650 bp for *cyt1Aa*.

Since ribosome display is an in vitro system, one of the challenges is to prevent the misfolding of the nascent protein, in particular those with disulfide bridges. It has been established that S30 extract from *E. coli* contains accessory protein, such as chaperones, that enable the correct folding and stability of displayed proteins during in vitro translation (Wörn and Plückthun 2001; Jermetus et al. 2001). The advantage to display Bt toxins in a toxic fragment format is the lack of disulfide bridges since cysteins residues are removed after toxin activation. To determine that Cry1Ac toxin is folded correctly we analyzed the binding ability of ternary complex to the receptor CR7-12 by ELISA binding assays. Figure 4c shows that ternary complex prepared with Cry1Ac mRNA specifically bound to CR7-12. Polyclonal antibodies anti-Cry1Ac and anti-Cyt1Aa or Cry11Aa toxin immobilized on ELISA-wells were also able to trap the ternary complex prepared with mRNA of Cry1Ac or Cyt1Aa toxins, respectively (Fig. 4c).

### Specific selection of Bt toxins by phage display and ribosome display

**Fig. 5:**
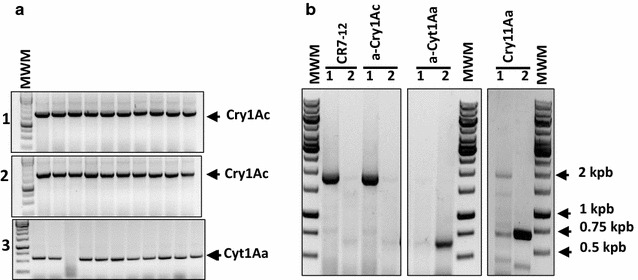
**a** Colony PCR after affinity selection of M13-toxins phage particles. A mixture of M13-Cry1Ac and M13-Cyt1Aa phages (1:1) was applied to immobilized cadherin fragment CR7-12 (*1*), anti-Cry1Ac antibody (*2*) and anti-Cyt1Aa antibody (*3*). *E. coli* XL1-Blue MRF’ cells were infected with the output phages and ten random colonies were analyzed by PCR to amplify *cry1Ac* or *cyt1Aa*. **b** RT-PCR products after selection of ternary complex prepared with a mixture of Cry1Ac and Cyt1Aa mRNA (1:1) against cadherin fragment CR7-12, anti-Cry1Ac antibody, anti-Cyt1Aa antibody or Cry11Aa toxin. *Lane 1* RT-PCR preformed with specific primers for Cry1Ac toxin; *lane 2* RT-PCR performed with specific primers for Cyt1Aa toxin. *MWM* Mass weight marker GeneRuler 1 kb DNA Ladder

The success of molecular display techniques relies on a physic link between the protein displayed (phenotype) and the coding-sequence (genotype). We showed the functional display of Bt toxins on M13 phages and ribosomes. To determine that we are able to recover the genetic information, input M13 phages displaying Cry1Ac or Cyt1Aa toxins were mixed 1:1 ration and selected against cadherin fragment CR7-12, anti-Cry1Ac antibody or anti-Cyt1Aa antibody. After selection, *E. coli* was infected with the output phages and ten clones were randomly selected to identify the *cry1Ac* or *cyt1Aa* genes from the phagemids by PCR amplification. Figure 5a shows that the ten clones analyzed presented a DNA fragment of 1.8 kb that corresponds to *cry1Ac* gene when the mixture of phages were selected against cadherin fragment CR7-12 or anti-Cry1Ac antibody. In the case where anti-Cyt1Aa antibody was used as bait, nine clones contained the fragment of 0.65 kb that corresponds to *cyt1Aa* gene. These data shows that we can recover specifically the Bt toxin gene with its cognate ligand used as bait for biopanning.

In the case of the toxins displayed on ribosomes, we carried out a similar scheme of selection with ternary complex prepared with 1:1 equimolar ration of Cry1Ac and Cyt1Aa mRNA. After affinity selection, the mRNA was eluted, reverse transcribed and the cDNA was used as template in a PCR reaction to amplify the *cry1Ac* or *cyt1Aa* gene. As was expected, mRNA of Cry1Ac toxin from the ternary complex was only recovered when cadherin fragment CR7-12 or antibody anti-Cry1Ac were used as bait. In the case of the mRNA of Cyt1Aa toxin was only recovered when the bait was the antibody anti-Cyt1Aa antibody or Cry11Aa toxin (Fig. 5b).

## Discussion

Although display in phages of Cry toxins has shown not to be optimal (Bravo et al. 2013), few examples of successful selection of improved variants of certain Cry toxins have been reported (Ishikawa et al. 2007; Craveiro et al. 2010; Oliveira et al. 2011). Nevertheless the display systems used are not likely to be efficient to display and select improved variants for most Cry toxins (Ishikawa et al. 2007; Craveiro et al. 2010; Oliveira et al. 2011). Therefore, the improvement of display systems that efficiently display Cry toxins allowing the creation of large libraries could provide means for rapid evolution of most Bt toxins.

In the case of phage display we show here that the display of two structurally non-related Bt toxins could be improved by changing the peptide leader sequence of the fusion protein, that relies on the Sec translocation pathway, for a peptide leader sequence that relies on the signal recognition particle pathway (SRP). Since the Sec pathway translocates proteins post-translationally while the SRP pathway co-translationally, this result suggests that Cry1Ac folding in the cytoplasm limits translocation and display efficiencies. Furthermore, we show that by using a helper phage (Phaberge) that assembles phage particle preferring the Cry1Ac-pIII fusion protein, display of Cry1Ac was further improved (Fig. 1a). More interestingly we show that Cry1Ac is properly folded retaining its receptor binding capacities and toxicity. Using the system described here we have successfully displayed Cry3Aa toxin that is toxic to coleopteran insects (Additional file 1: Figure S1). The phage display system described here allows great flexibility for panning selection since the different display levels of Cry toxins could be used for selection of variants with different binding affinities. Enhanced display of Bt toxin libraries using Phaberge and SRP translocation sequence are likely to select binders with moderate affinity due to the selection of binders by avidity since several binding sites are present in one phage particle. Consecutive mutagenesis and selection using display systems that allow moderate (SRP sequence and M13) or low (Sec sequence and M13) display efficiencies would select binders with higher binding affinities. We believe that depending on the binding target (receptor vs insect gut membranes) and toxicity of the native Cry toxin used for library construction the phage display systems described here could be useful to put in place a panning procedures to select improved toxins.

Ribosome display has the unique feature that it allows the construction of large libraries that are more likely to be useful to select toxins with improved binding and toxicity. We show here that Cry1Ac toxin of 60 kDa is efficiently displayed in ribosomes and that selection with a cadherin receptor specifically selected Cry1Ac-displayed ribosomes. To our knowledge this is the first example of a protein of more than 50 kDa that is successfully displayed in ribosomes. It still needs to be determined if other 3D-Cry toxins of similar size could be displayed on ribosomes to determine if ribosome-display could be a general system for evolution of other 3D-Cry toxins. However, it is still possible to display individual domains II or III that are involved in receptor binding for library construction and selection of improved binders using ribosome display. In this regard, we have successfully displayed Cry1Ac domain II and shown that it retains cadherin receptor binding (Additional file 1: Figure S2).

In the case of Cyt1Aa we show that is can be efficiently displayed using both the improved phage display system described here and also using ribosome display. Nevertheless, Cyt1Aa was capable of Cry11Aa binding only when displayed in ribosomes (Fig. 5b) in contrast when displayed in M13 where it was not capable to bind Cry11Aa (data not shown). As mentioned earlier, Cyt1Aa enhances the toxicity of Cry11Aa or Cry4Ba against mosquitoes by functioning as a surrogate receptor. We will use ribosome display to select Cyt1Aa mutants that bind other Cry toxins as a way to develop tools for improving Bt Cry toxicity against specific insect targets. In the case of Cyt2Aa toxin it was shown that insertion of a peptide sequence with aphid APN binding activity in certain loop regions resulted in Cyt2Aa toxins with aphid toxicity (Chougule et al. 2012). Thus, by using both display systems described here, selection of Cyt1Aa variants capable to bind to insect gut membranes of certain insect species could select Cyt1Aa toxins with toxicity to certain insect pests.

Overall we describe here two efficient display systems for Bt toxins that are likely to be useful to evolve Bt toxicity to control pets that show low susceptibility to these toxins or to control insect pests that evolved resistance to Cry toxins in field conditions.

## Additional file

10.1186/s13568-015-0160-1 Western-blot analysis of M13-Cry3Aa. **Figure S2**. Analysis of domain II of Cry1Ac displayed on ribosomes.
