# Supplementary material for: Improvement and efficient display of Bacillus thuringiensis toxins on M13 phages and ribosomes
Source: AMB Express. 2015 Nov 25;5:73. doi: 10.1186/s13568-015-0160-1 (PMC4659786; doi:10.1186/s13568-015-0160-1)
Supplement: Supplementary file 1 — 10.1186/s13568-015-0160-1 Western-blot analysis of M13-Cry3Aa. Figure S2. Analysis of domain II of Cry1Ac displayed on ribosomes. [file 13568_2015_160_MOESM1_ESM.pdf]

Supplementary material

Improvement and efficient display of *Bacillus thuringiensis* toxins on M13 phages and ribosomes

Sabino Pacheco[1], Emiliano Cantón[1], Fernando Zuñiga-Navarrete[1], Frédéric Pecorari[2], Alejandra Bravo[1] and Mario Soberón[1*]

[1]Instituto de Biotecnología, Universidad Nacional Autónoma de México. Apdo. postal 510-3, Cuernavaca 62250, Morelos, Mexico.

[2]Institut de Recherche en Santé de l'Université de Nantes. INSERM U892 - CNRS 6299 – CRCNA, 8 quai Moncousu, BP 70721, 44007 Nantes, Cedex 1, France.

*Corresponding author: mario@ibt.unam.mx

tel 527773291618

fax 527773291624

Figure S1

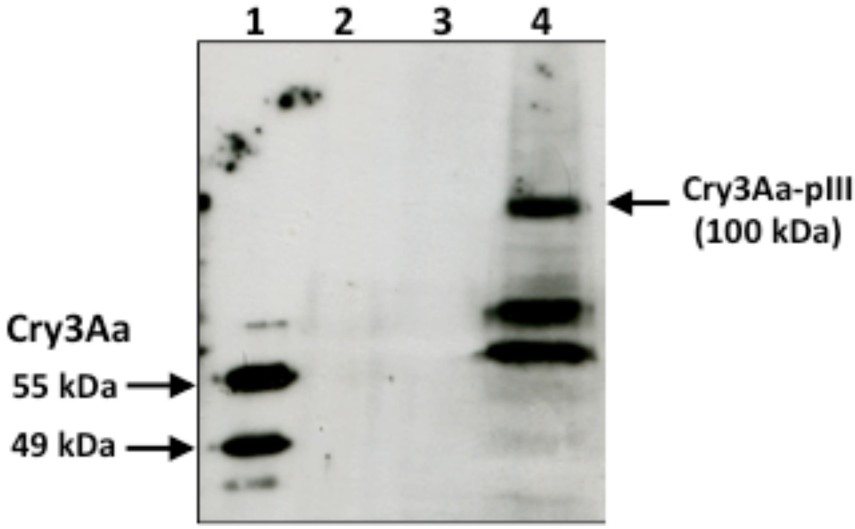

Figure S1. Western-blot analysis of M13-Cry3Aa. The *cry3Aa* gene was PCR-amplified from *Bt subsp. tenebrionis* with the primers 3Aup and 3Alow and ligated into the phagemid pCADS. *E. coli* HB2151 was transformed with the phagemid pCADS-Cry3Aa and phages were produced. Lane 1, Chymotrypsin actived Cry3Aa toxin. Lane 2, Helper phage. Lane 3. Phage particles prepared with VCSM13 helper phage. Lane 4, Phage particles prepared with Phaberge.

Figure S2

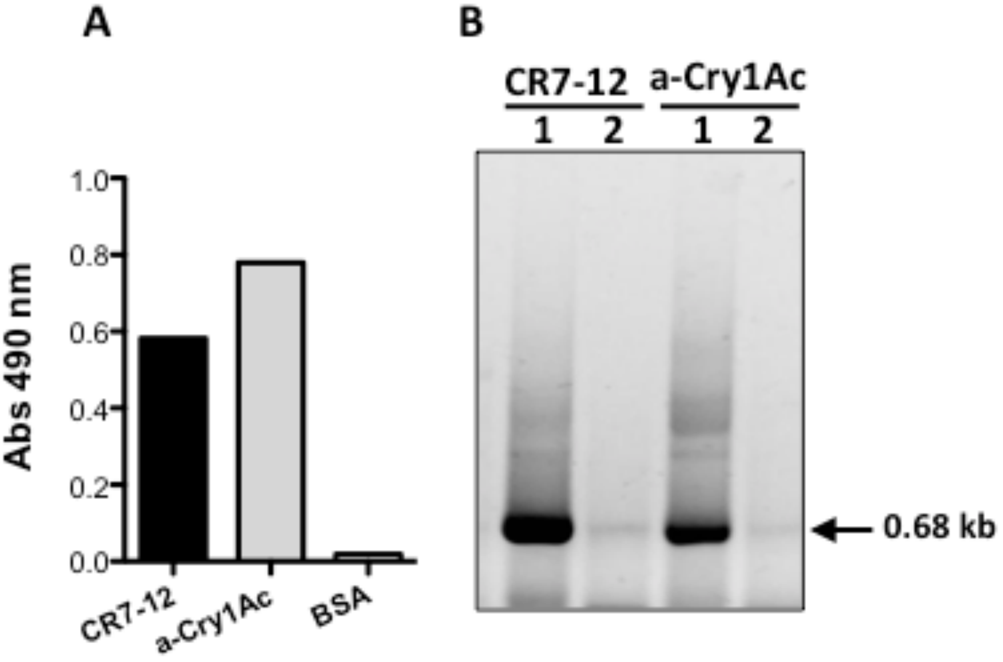

Figure S2. Analysis of domain II of Cry1Ac displayed on ribosomes. Fragment of domain II was PCR-amplified with the primers FDIIc and RDIIc and ligated into the vector pFPRDV. (A) Binding assay of ternary complex prepared with mRNA of domain II to cadherin fragment CR7-12, antibody anti-Cry1Ac and BSA. (B) Ternary complex prepared with an equimolar mixture of mRNA (1:1) of domain II and Cyt1Aa were summited to affinity selection against cadherin fragment CR7-12 and antibody anti-Cry1Ac. After selection mRNA eluted was reverse transcripted and PCR amplified with the primers SDMRGS/RDIIc (lane1) or SDMRGS/CytRRD (lane2).
